# Morphological Species Delimitation in The Western Pond Turtle (*Actinemys*): Can Machine Learning Methods Aid in Cryptic Species Identification?

**DOI:** 10.1093/iob/obae010

**Published:** 2024-04-02

**Authors:** R W Burroughs, J F Parham, B L Stuart, P D Smits, K D Angielczyk

**Affiliations:** Department of Ecology and Evolution, Stony Brook University, Stony Brook, NY 11794, USA; Center for Inclusive Education, Stony Brook University, Stony Brook, NY 11794, USA; Department of Geological Sciences, California State University, Fullerton, CA 92834, USA; Section of Research and Collections, NC Museum of Natural Sciences, Raleigh, NC 27601, USA; 952 NW 60th St., Seattle, Washington, WA 98107, USA; Negaunee Integrative Research Center, Field Museum of Natural History, Chicago, IL 60605, USA

## Abstract

As the discovery of cryptic species has increased in frequency, there has been an interest in whether geometric morphometric data can detect fine-scale patterns of variation that can be used to morphologically diagnose such species. We used a combination of geometric morphometric data and an ensemble of five supervised machine learning methods (MLMs) to investigate whether plastron shape can differentiate two putative cryptic turtle species, *Actinemys marmorata* and *Actinemys pallida. Actinemys* has been the focus of considerable research due to its biogeographic distribution and conservation status. Despite this work, reliable morphological diagnoses for its two species are still lacking. We validated our approach on two datasets, one consisting of eight morphologically disparate emydid species, the other consisting of two subspecies of *Trachemys* (*T. scripta scripta, T. scripta elegans*). The validation tests returned near-perfect classification rates, demonstrating that plastron shape is an effective means for distinguishing taxonomic groups of emydids via MLMs. In contrast, the same methods did not return high classification rates for a set of alternative phylogeographic and morphological binning schemes in *Actinemys*. All classification hypotheses performed poorly relative to the validation datasets and no single hypothesis was unequivocally supported for *Actinemys*. Two hypotheses had machine learning performance that was marginally better than our remaining hypotheses. In both cases, those hypotheses favored a two-species split between *A. marmorata* and *A. pallida* specimens, lending tentative morphological support to the hypothesis of two *Actinemys* species. However, the machine learning results also underscore that *Actinemys* as a whole has lower levels of plastral variation than other turtles within Emydidae, but the reason for this morphological conservatism is unclear.

## Introduction

The goal of conservation biology is to help recognize and preserve biodiversity in the face of a constantly changing global environment, with particular emphasis on species and environments that are under direct and constant threat from anthropogenic factors (e.g., Soulé 1985; Hunter et al. 2021). However, efforts at mitigating the impacts of anthropogenic climate change and other environmental threats are hampered by many factors (e.g., political, societal, and scientific). Among the scientific factors is the challenge of being able to define and differentiate species. Traditionally, the majority of species were diagnosed on the basis of their morphological characteristics (e.g., coloration, size, shape, and discrete morphological traits) (Padial et al. 2010). Although this approach works well for morphologically distinct species, it can be a difficult task to differentiate closely related species from one another. In particular, this can be a problem between cryptic species (e.g., Fišer et al. 2018) that may, at least initially, only be distinguished by genetic differentiation and have few or no apparent outward morphological characteristics that can be used for identification. In other cases, speciation may be ongoing and genetic differentiation may be relatively small, leading to general confusion about the taxonomic status of populations or groups. Having morphological characteristics that can distinguish between groups can help resolve these problems. Thus, techniques that facilitate the identification of subtly divergent morphologies to distinguish species are of great interest to taxonomists, conservation biologists, and biogeographers, among others. A growing field of biological computing is the application of machine learning methods (MLMs) to multivariate trait data in attempts to morphologically identify species (e.g., Baylac et al. 2003; Dobigny et al. 2003; MacLeod 2007; Van Boxclaer and Schultheiß 2010; van den Brink and Bokma 2011; Doyle et al. 2018; Monson et al. 2018, Quenu et al. 2020; Derkarabetian et al. 2022). Here, we present a study that applies MLMs to geometric morphometric data in an attempt to distinguish between the northwestern pond turtle (*Acitnemys marmorata*) and southwestern pond turtle (*Actinemys pallida*), which were recently proposed to be distinct species on the basis of genetic data (Spinks et al. 2014).

### *Actinemys* morphological variation and alpha taxonomy

Unlike most other emydine species, which are characterized by highly disparate phenotypes (e.g., Bonin et al. 2006), species of *Actinemys* show little morphological variation across its range, and different interpretations of this variation have emerged over the past seven decades. Seeliger (1945) divided *A. marmorata* (as *Clemmys marmorata*) into two named subspecies: *Clemmys marmorata marmorata* in the northern part of the species’ range and *Clemmys marmorata pallida* to the south. She differentiated the northern subspecies, *C. m. marmorata* from *C. m. pallida* by the presence of a pair of triangular inguinal scales and darker neck markings in the former, although small inguinal scales are sometimes present in *pallida* and Seeliger recognized an intergrade zone of these two subspecies in central California. Seeliger did not formally include the Baja California populations in either taxon, implying the existence of a third distinct but unnamed subspecies.

Holland (1992) performed a morphometric analysis of carapace shape of *A. marmorata* (as *C. marmorata*) specimens from three geographic areas. He concluded that geographic distance was a poor indicator of morphological differentiation, and instead hypothesized that geographic features such as breaks between different drainage basins are probably more important barriers to dispersal and gene flow. Additionally, Holland (1992) suggested that morphological differences were more pronounced as the magnitude of barriers and distance increased, but this variation required many variables to adequately capture, implying only very subtle morphological differentiation between putatively distinct populations. Holland concluded that *A. marmorata* should be divided into three distinct species: a northern species, a southern species, and a Columbia Basin species, but he left these species unnamed.

Following Holland's (1992) study, work on morphological variation in *A. marmorata* has focused primarily on differentiation between populations over a portion of the species’ total range. Most of these studies considered how local biotic and abiotic factors may contribute to differences in carapace length, and they found that size can vary greatly between different populations (Lubcke and Wilson 2007; Germano and Rathbun 2008; Germano and Bury 2009; Bury et al. 2010; Manzo et al. 2021). There also has been interest in size-based sexual dimorphism in *A. marmorata*, with males being larger on average than females based on total carapace length and other linear measurements, although the quality of size as a classifier of sex can vary greatly between populations (Lubcke and Wilson 2007; Germano and Rathbun 2008; Germano and Bury 2009; also see Holland 1992). So far, morphological studies have not recovered morphological variation that corresponds to the clades recovered by genetic studies of the *A. marmorata* complex (Spinks and Shaffer 2005; Spinks et al. 2010; Spinks et al. 2014).

Our geometric morphometric analysis of shell shape will test species boundaries proposed by the genetic studies by determining which, if any, of the genetic groupings are morphologically diagnosable. This work is a crucial follow up to genetic studies of rangewide variation because recent research shows that multispecies coalescent analyses may over-split species (Chambers and Hillis 2020; Cadena and Zapata 2021; Smith and Cartsens 2022). It is critical to look for congruence among methods and data types such as life history, geographical distribution, morphology, and behavior when approaching species (Knowles and Carstens 2007; Schlick-Steiner et al. 2010; Carstens et al. 2013). In the case of the distinctiveness of *A. marmorata* and *A. pallida*, the genetic data sets are not congruent (Spinks and Shaffer 2005; Spinks et al. 2010; Spinks et al. 2014), there are few phenotypic data to support this split, and life history, geographical, or behavioral justifications for the species boundary are so far lacking.

### Machine learning and morphometrics

Machine learning is an extension of known statistical methodology that emphasizes predictive accuracy and generality, often at the expense of the interpretability of individual parameters (Hastie et al. 2009; Goodfellow et al. 2016; Greener et al. 2022). Basic statistical approaches are supplemented by randomization, sorting, and partitioning algorithms, along with the maximization or minimization of summary statistics, in order to best estimate a general model for all data, both sampled and unsampled (Hastie et al. 2009; Goodfellow et al. 2016; Greener et al. 2022). Machine learning approaches have been used in medical research, epidemiology, economics, and automated identification of images, and there is increasing interest in the potential of machine learning in the context of morphometric analysis for identification of cryptic species (e.g., MacLeod 2017; Courtnenay et al. 2019; Püschel et al. 2018; Pyron 2023).

There are two major classes of MLMs: unsupervised and supervised learning. Unsupervised learning methods are used with unlabeled data where the underlying structure is estimated; they are analogous to clustering and density estimation methods (Kaufman and Rousseeuw 1990). Supervised learning methods are used with labeled data where the final output of data is known and the rules for going from input to output are inferred. These are analogous to classification and regression models (Breiman et al 1984; Hastie et al. 2009).

Geometric morphometrics has become a standard means to capture fine-scale shape variation in a variety of contexts (e.g., Claude 2008; Lawing and Polly 2010; Zelditch et al. 2012; Cooke and Terhune 2014; Mitteroecker and Schaefer 2022), and one application of the method is to identify variation that can be used for diagnosing cryptic species. This approach has the potential to make the task of identifying and maintaining endangered or conserved groups much easier, and can contribute to improved classifications of extinct taxa and populations. Geometric morphometric approaches to identifying differences in morphological variation between classes, including cryptic species, have often relied on methods like linear discriminant analysis (LDA) and canonical variates analysis (CVA) (Polly 2003, 2007; Valenzuela et al. 2004; Francoy et al. 2009; Sztencel-Jabłonka et al. 2009; Edwards et al. 2011; Mitrovski-Bogdanovic et al. 2013; Dillard 2017). Because of their similarity to multivariate approaches like principal components analysis (PCA), these methods are comparatively straightforward ways of understanding the differences in morphology between classes. They also benefit from producing results that can be easily visualized, which aids in the interpretation and presentation of data and results. However, these approaches usually require an *a priori* classification to be in place, with tests focusing on how well specified groups in the classification can be differentiated using shape data, instead of assessing which among a set of alternative classification hypotheses is optimal. For example, studies such as those of Caumul and Polly (2005) and Polly (2007) focused on comparing different aspects of morphology and their fidelity to a classification scheme instead of comparing the fidelity of one aspect of morphology to multiple classification schemes. The study of Cardini et al. (2009) is noteworthy in this context because they compared morphological variation in marmots at the population, regional, and species levels and determined the fidelity of shape to divisions at each of these levels. Machine learning methods have been combined with geometric morphometric data to study shape variation in a variety of contexts, including automated taxon identification and classification of groups (Baylac et al. 2003; Dobigny et al. 2003; MacLeod 2007; Van Boxclaer and Schultheiß 2010; van den Brink and Bokma 2011; Navega et al. 2015; Kutlu et al. 2017; Doyle et al. 2018; Quenu et al. 2020; Wöber et al. 2021; Páramo et al. 2022; Moclan et al. 2023), and an advantage of these methods is that they provide more flexibility in comparing alternative classification hypotheses.

### This study

In our study, we analyzed a set of geometric morphometric data that captures plastron shape with an ensemble of supervised MLMs to compare the congruence of morphological data to different classification hypotheses proposed for the *A. marmorata* complex. Each of the MLMs we used has different advantages for understanding how to classify our specimens, and congruence between them increases support for the preferred conclusion (Hastie et al. 2009). We focus on plastron shape for multiple reasons. First, it is relatively simple to collect geometric morphometric data on plastron shape from two-dimensional photographs because the structure is virtually flat. This approach allows both museum specimens and individuals photographed in the field to be analyzed together. Second, previous work has suggested that there are strong differences in plastron shape among traditionally recognized emydine species (Claude 2006; Angielczyk et al. 2011; Angielczyk and Feldman 2013). Third, a large dataset was readily available due to these previous studies and additional work examining the effects of tectonic deformation on geometric morphometric datasets (Angielczyk and Sheets 2007). Fourth, variation in aspects of plastron phenotype, such as shape and marking patterns has been shown to facilitate very fine-scale discrimination among turtles at or below the species level, such as differentiation of individuals from separate nesting sites (Myers et al. 2006) or the identification of individuals in recapture studies and conservation efforts (Tichý and Kintrová 2010; Cooley et al. 2013; Cross et al. 2014; Suriyamongkol and Mali 2018).

## Material and methods

### Nomenclature

The generic status of the western pond turtle has long been unsettled. Phylogenetic evidence resulted in strong consensus that it should not be placed in the genus *Clemmys* as historically recognized (e.g., Seeliger 1945; Holland 1992), but considerable disagreement has since ensued on the alternate use of *Emys* or *Actinemys*. Some authors preferred to use an inclusive *Emys* to emphasize the relatedness of *marmorata* (Baird and Girard 1852) to other coordinate lineages in the genus *Emys* (e.g., *Emys blandingii, Emys orbicularis*), whereas others preferred recognizing three monotypic genera (*A. marmorata, Emydoidea blandingii, Emys orbicularis*). The arguments primarily involve appeals to morphological disparity (Holman and Fritz 2001; Fritz et al. 2011) or a preference to avoid monotypic genera and to apply a widely known name to a distinctive and well-supported clade (Feldman and Parham 2002; Parham and Feldman 2002; Spinks and Shaffer 2009; Spinks et al. 2016). Spinks et al. (2016) showed that the *Emys* [sensu lato] clade is recovered as monophyletic by analyses that use mitochondrial or nuclear DNA markers.

In recent years, the three-genus scheme has become more prevalent. This is partially because of studies that proposed naming new species from parts of *Emys orbicularis* and *A. marmorata*, thereby mitigating the concerns about monotypic genera. Fritz et al. (2005) proposed splitting the populations of the European pond turtle (*Emys orbicularis*) on Sicily into a new species (*Emys trinacris*), although the species status of *Emys trinacris* was not accepted by some later authors (Spinks and Shaffer 2009; Speybroek et al. 2020). Later, Spinks et al. (2014) proposed that the western pond turtle (*Emys marmorata*) should be divided into two species, with southern populations being referred to as the species *Emys pallida* (an elevated subspecies name). Whether *Emys trinacris* or *A. pallida* are valid species is not settled, but given the clear preference for the three genus scheme for the *Emys* [sensu lato] clade, we will use the less inclusive genus *Actinemys* for *A. marmorata* and *A. pallida*.

The three-genus scheme results in a distinctive and well-supported clade (the *Emys* [sensu lato] clade of Feldman and Parham [2002]) being left without a name. Aside from the genus *Emys*, no previous name is available for this clade (Seidel and Ernst 2017). We rectify this by phylogenetically defining a new name below. We refrain from providing definitions for the genera *Emys, Emydoidea*, and *Actinemys* because they may have undergone ancient hybridizations (Spinks and Shaffer 2009) that would complicate the construction of phylogenetically defined names.

***Emysia*** Burroughs, Parham, Stuart, Smits, and Angielczyk, 2024, New Clade Name
**Registration number—**1008
**Definition—**The smallest clade that includes *Emys orbicularis*  Linneaus 1758, *Emyd.* (orig. *Cistuda*) *blandingii* (Holbrook 1838), *A.* (orig. *Emys*) *marmorata* (Baird and Girard 1852) but not *Terrapene* (orig. *Testudo*) *carolina*  Linneaus 1758, *Glyptemys* (orig. *Testudo*) *insculpta* (Le Conte 1830), or *C.* (orig. *Testudo*) *guttata* (Schneider 1792).
**Reference Phylogeny—** Spinks et al. (2016, Fig. 2).
**Composition—** *Emysia* includes the extant species of emydine turtles that were placed in the genus *Emys* by Feldman and Parham (2002) and followed by some later authors (see Seidel and Ernst 2017 for a review) as well as species arising from later divisions (e.g., *A. pallida*). *Emysia* also includes extinct chronospecies of the genus *Emys* (Fritz 1998).
**Comments—**Although this clade has been identified for over 20 years (Holman and Fritz 2001; Feldman and Parham 2002), the only name assigned to it has been the genus *Emys* (Fedman and Parham 2002; Seidel and Ernst 2017).
***Pan-Emysia*** Burroughs, Parham, Stuart, Smits, and Angielczyk, 2024, New Clade Name
**Registration number—**1009
**Definition—**The total crown clade of Emysia.
**Reference Phylogeny—** Spinks et al. (2016, Fig. 2).
**Composition—**The known composition is identical to that of *Emysia* (above).
**Comments—**None.

### Specimens sampled

We assembled three geometric morphometric datasets describing turtle plastron variation for this analysis: 1) specimens from seven distinct emydine species and an outgroup species from the sister group Deirochelyinae; 2) *Trachemys scripta* specimens from two subspecies (*T. s. elegans, T. s. scripta*); and 3) *A. marmorata* sensu lato specimens from across the complex's geographic range. The first two datasets are intended to validate that machine learning techniques can differentiate species-level groupings of emydine turtles using plastron shape. We expect that the first case represents a low complexity dataset because of the high level of plastron shape disparity that exists among these species and their relatively deep phylogenetic divergences (Claude et al. 2003; Claude 2006; Angielczyk et al. 2011; Spinks et al. 2016), whereas the second dataset should be relatively higher in complexity and more analogous to the *A. marmorata* example because of the close phylogenetic relationship and likely intergradation between the *Trachemys* subspecies (e.g., Parham et al. 2020; Vamberg et al. 2020). We predict that the *A. marmorata* dataset should be of the highest complexity and our greatest challenge given the finding that only very subtle differences exist between geographically distinct populations (Holland 1992).

The first dataset (“emydines”) we analyzed included 992 total specimens from seven Emydinae species (*C. guttata, Emyd. blandigii, E. orbicularis, G. insculpta, G. muhlenbergii, T. coahuila, T. ornata*) and one outgroup species (*Chrysemys picta*) from Deirochelyinae, the clade that with Emydinae forms Emydidae. These specimens were used in the analyses of Angielczyk et al. (2011) and Angielczyk and Feldman (2013), and for each species include a mix of males and females ranging in size from hatchlings to large adults. The second dataset (*Trachemys*) is a compilation of 101 specimens of two subspecies of the deirochelyine *T. scripta*: 51 specimens of *T. s. scripta* and 50 specimens of *T. s. elegans*. These landmark data are new to this study, and include a mix of male and female adult specimens. The final dataset (marmorata) consisted of 532 adult *A. marmorata* museum specimens that comprise a subset of those included in previous studies (Angielczyk and Sheets 2007; Angielczyk et al. 2011; Angielczyk and Feldman 2013). All three landmark datasets are available at the Dryad Digital Data Repository (Burroughs et al. 2024).

### *Actinemys* sample binning

Because the previous molecular phylogenetic studies of *A. marmorata* did not use vouchered specimens (Spinks and Shaffer 2005; Spinks et al. 2010; 2014), we were not able to directly sample the individuals in their studies. Instead, our specimen classifications were based solely on the geographic occurrence information associated with the specimens and not explicit assignment using molecular data. For each taxonomic hypothesis, specimens were assigned to one of the possible classes based on geographic occurrence data recorded in museum collections. In cases where precise latitude and longitude information were not available, we estimated them from other locality information. The exact barriers between different biogeographic regions are unknown and unclear, so we represented each hypothesis with multiple possible realizations reflecting the classification uncertainty for specimens present at the geographic boundaries.

**Fig. 1:**
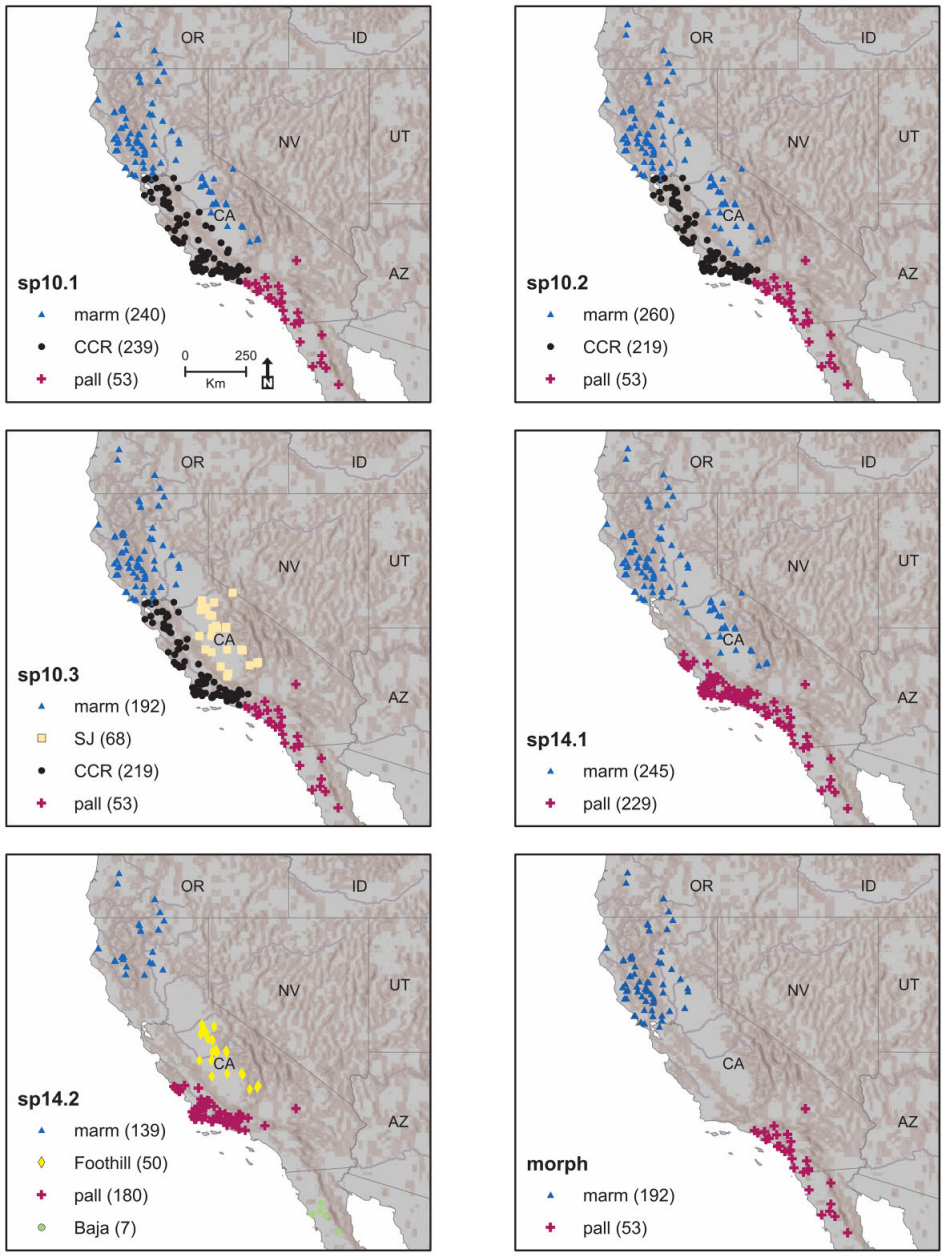
Geographic distribution of specimens used for comparing the hypothesized subdivisions of the *A. marmorata* complex. Each hypothesized scheme has two or more possible classes (see Table 1 for explanation of schemes). Sample size differs between schemes because of variance in our ability to confidently assign museum specimens to the schemes. The number of localities shown on each map is less than the number of specimens sampled because some localities produced multiple specimens. *A. marmorata*, marm; *A. pallida*, pall; central coast ranges, CCR; San Joaquin valley, SJ; Baja peninsula, Baja; Sierra foothills, Foothill.

We used three main binning schemes for the *A. marmorata* dataset (Fig. 1, Table 1). Our first binning scheme (SP10) consisted of three permutations based on the phylogeographic hypotheses of Spinks et al. (2010). All three permutations included a class for *A. marmorata* specimens from northern populations (marm) as well as a class for those assigned to *A. pallida* (pall) and an intergrade zone in the Central Coast Ranges (CCR). The permutations differed in the assignment of samples from the San Joaquin Valley (Fig. 1). In permutations SP10.1 and SP10.2, specimens from the western San Joaquin Valley were assigned to either CCR or marm, reflecting uncertainty regarding their genetic affinity as explained above. In SP10.3, these specimens were assigned to a San Joaquin class reflecting the mitochondrial distinctiveness shown by Spinks and Shaffer (2005). Our second binning scheme (SP14) included two permutations based on the results of Spinks et al. (2014). SP14.1 was based on their phylogenetic network analysis and SP14.2 was based on their Bayesian species delimitation analysis. The latter permutation required the addition of two new classes, “Baja” and “Foothill,” to accommodate the genetic groupings recovered by the single nucleotide polymorphism (SNP) structure analysis that was used by Spinks et al. (2014) to create the guide tree for the species delimitation analysis using the software package BPP (Yang and Rannala 2010; Yang 2015). Finally, we proposed a conservative morphological hypothesis (Morph) to compare the molecular hypotheses with an alternative approximating the original taxonomic hypothesis for the group. This scheme is comprised solely of the marm and pall classes from the SP10.3 permutation.

**Table 1:** Details of binning schemes used for the *A. marmorata* complex.

Abbreviation	Number of classes	Reference
SP10.1	3	Spinks et al. (2010)
SP10.2	3	Spinks et al. (2010)
SP10.3	4	Spinks et al. (2010)
SP14.1	2	Spinks et al. (2014)
SP14.2	4	Spinks et al. (2014)
Morph	2	Spinks et al. (2010)

### Morphometric landmarks

**Fig. 2:**
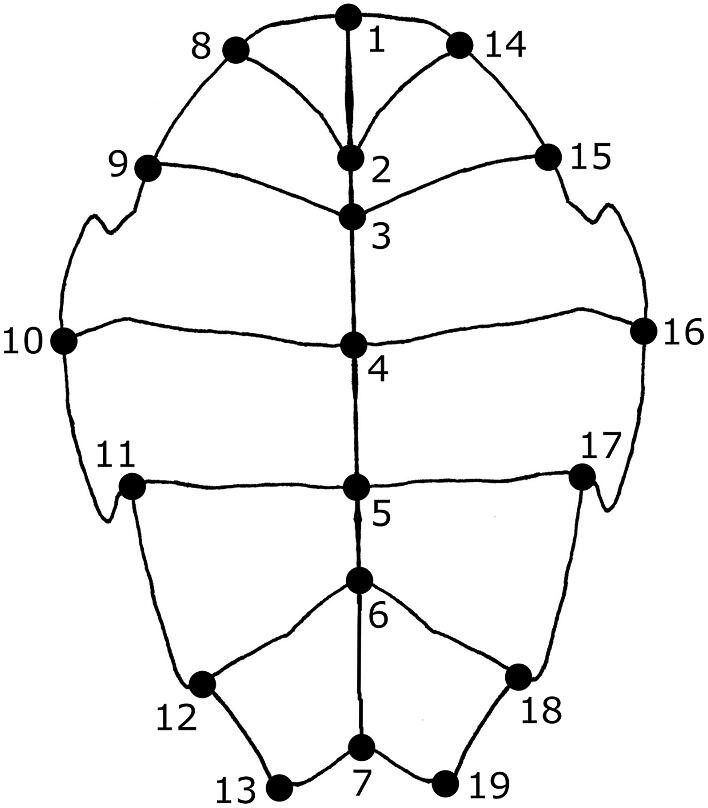
Depiction of general plastron shape of *A. marmorata* and position of the 19 landmarks used in this study. Anterior is toward the top of the figure.

Following previous work on plastron shape (Angielczyk and Sheets 2007; Angielczyk et al. 2011; Angielczyk and Feldman 2013), we used TpsDig 2.04 (Rohlf 2005) to digitize 19 two-dimensional landmarks (Fig. 2). Seventeen of the landmarks are at the endpoints or intersections of the keratinous scutes that cover the plastron. Twelve of the landmarks were symmetrical across the axis of symmetry. Because damage prevented the digitization of all the symmetric landmarks in some specimens, we reflected landmarks across the axis of symmetry (i.e., midline) prior to analysis and used the average position of each symmetrical pair. In cases where damage or incompleteness prevented both symmetric landmarks from being digitized, we used only the single member of the pair that was present. We conducted all subsequent analyses on the resulting “half” plastra. We did not use semi-landmarks to attempt to capture the shape of the outline of the plastron, but our landmark configuration should still capture an adequate amount of shape variation for the problem at hand considering that a near identical configuration was able to differentiate *T. scripta* individuals from nesting sites less than 5 km apart (Myers et al. 2006).

We superimposed the plastral landmark configurations using generalized Procrustes analysis (Dryden and Mardia 1998), after which we calculated the principal components (PC) of shape using the shapes package for R (Dryden 2013; R Core Team 2016). All specimens were used for superimposition, after which the subset labeled for each of the schemes were used in model training and testing (see below).

### Digitization error

We estimated the possible effect of digitization error (Arnqvist and Mårtensson 1998; von Cramon-Taubadel et al 2007; Muñoz-Muñoz and Perpinan 2010) on our results by comparing within-specimen (replicated) Procrustes distances using a one-way Procrustes analysis of variance (ANOVA). In this case, we chose a single specimen (CAS 1236) and had the same author who conducted the initial landmark placement (KDA) re-digitize the landmarks five times on that specimen.

### Sexual dimorphism

*Actinemys marmorata* is known to display sexual dimorphism in plastron shape, particularly the presence of a plastral concavity in males (Seeliger 1945). To test for biases resulting from sexual dimorphism in our *A. marmorata* dataset, we performed a CVA. This analysis attempts to maximize linear distance between groups (Male, Female), determine if there is a significant difference between the mean of groups (using Hotelling's *T*-Squared test), and determine if there is a significant difference in the distance between the mean of groups (Mahalanobis Distance). Sex-ratios for each binning scheme are presented in Table 2.

**Table 2:** Within-class sex ratios of the *Actinemys* complex for each binning scheme used in this study. Sex ratios are presented as female/male. Central coast ranges, CCR; San Joaquin valley, SJ; Baja peninsula, Baja; Sierra foothills, Foothill.

Scheme	Sex ratios
SP10.1	CCR: 53/48; Marm: 87/97; Pall: 18/23
SP10.2	CCR:50/40; Marm: 90/105; Pall: 18/23
SP10.3	CCR: 50/40; Marm: 74/82; Pall: 18/23; SJ: 16/23
SP14.1	Marm: 87/100; Pall: 54/51
SP14.2	Baja 0/5; Foothill: 13/15; Marm: 53/50; Pall: 36/31
Morph	Marm: 74/81; Pall: 18/23

### Inguinal scales

One of the key morphological characters used by Seeliger (1945) to differentiate *A. m. marmorata* from *A. m. pallida* is the presence and size of the inguinal scales. This character has subsequently been used in the differential diagnosis of *A. marmorata* and *A. pallida* (Spinks et al. 2014), despite the fact that the character is known to be highly variable (Seeliger 1945; Buskirk 1990). To investigate whether a shape difference exists between specimens with and without inguinal scales, we assessed the presence or absence of inguinal scales for the full *Actinemys* dataset using our photographs of specimens. In some cases, the presence or absence of inguinal scales could not be determined because of issues such as the position of the hind limbs in the photo or damage to the shell, and these specimens were excluded from the analyses considering inguinal scales. In total, we compiled data for 425 specimens, 241 with inguinal scales and 184 without. To determine if there is a significant difference between the mean of the groups with and without inguinal scales, we ran a CVA with the same interpretation of results as described above for sexual dimorphism. Ratios of specimens with and without inguinal scales for each of the binning schemes are presented in Table 3.

**Table 3:** Within-class ratios of *Actinemys* specimens with or without inguinal scales for each binning scheme used in this study. Ratios are presented as inguinal present/inguinal absent. Central coast ranges, CCR; San Joaquin valley, SJ; Baja peninsula, Baja; Sierra foothills, Foothill.

Scheme	Presence/absence of inguinal scales
SP10.1	CCR: 49/132; Marm: 180/18; Pall: 12/34
SP10.2	CCR:37/131; Marm: 192/19; Pall: 12/34
SP10.3	CCR: 37/131; Marm: 155/11; Pall: 12/34; SJ: 37/8
SP14.1	Marm: 184/18; Pall:38/145
SP14.2	Baja 4/3; Foothill: 26/7; Marm: 104/3; Pall: 26/114
Morph	Marm: 154/11; Pall: 12/34

### Habitat variables

Intraspecific variation in shell shape associated with water flow regime is well documented in aquatic emydid turtles (Rivera 2008; Rivera and Stayton 2011; Selman 2012; Ennen et al. 2014; Rivera et al. 2014), including *A. marmorata* (Lubcke and Wilson 2007), with individuals living in faster-flowing bodies of water tending to have lower, more streamlined shells. The *A. marmorata* complex occurs from sea level to over 2000 m elevation (Stebbins 2003; Bury et al. 2012) in bodies of water ranging from lotic fast-flowing streams to lentic ponds and wetlands. Therefore, there is a reason to suspect that individuals from different parts of the geographic range might display consistent differences in shell shape reflecting flow regime independent of their phylogenetic relationships. To test this hypothesis, we compiled data for two proxies of flow regime for our specimens; elevation and slope, using Quantum GIS (QGIS v.3.4 Maderia; see GIS Supplemental for workflow). We also compiled direct measurements of flow rate for a more limited number of specimens (*n* = 256).

To estimate elevation for each point, we extracted the mean elevation estimate from digital elevation models contained within the Global Multi-resolution Terrain Elevation Data 2010 dataset. Two raster maps were required to cover the entire geographic extent of the specimens sampled here (one with NW Corner Latitude of 30ºN and NW Corner Longitude of 120ºW and one with NW Corner Latitude of 30ºN and NW Corner Longitude of 150ºW), each having a resolution of 7.5-arc-seconds (approximately 250 m). We estimated slope as the angle of inclination in degrees with values between 0 and 90.

Using our geographic sampling as a range, we extracted water flow values from National Hydrology Dataset Plus (NHDPlus) GIS datasets. Currently, only sections 1801 and 1806 of NHDPlus overlap with the geographic range of our sample, meaning that flow data are available for only a subset of specimens. Flow values in NHDPlus datasets are estimated as maximum runoff flow (in feet per second) based on historical stream gauge data from 1971 to 2000. Due to minor amounts of error in georeferencing and the fact that turtle specimens are most often found adjacent to but not directly in water, we had zero points of direct overlap between turtles and bodies of water. Therefore, it was necessary to create a buffer zone surrounding our geolocated specimens. Buffer zones were created as 0.0015º circles (approximately 90 m in diameter). Next values of flow were extracted where buffered specimen localities and flow points intersected. Finally, because there were a number of identical flow values for any given buffered specimen locality, unique values for flow were extracted by specimen number. This final step gave a table of unique flow rates for each given specimen buffer zone. Rates were averaged for specimens with more than one flow rate value.

Once the elevation, slope and flow rate data were compiled, we conducted multivariate regressions of Procrustes coordinates versus the following independent variables: log altitude, log slope, and (untransformed) flow rate. Significance of the regression models was tested against a null hypothesis of complete independence between shape and the flow regime proxies, with proxy values being randomly assigned to the shape data 10,000 times.

### Supervised machine learning

In total, we used five different MLMs to investigate our different classification schemes: support vector machine (SVM), LDA, naiveBayes, decision tree classification (TREE), and random forests. We anticipate that multivariate data will cause some models to perform better than others, and therefore we used an ensemble approach (rather than a single ML algorithm) where we evaluated multiple models to determine if a consensus among models exists. We used the PC scores as variables for MLMs. For training datasets, we assigned species designations based on different biogeographic hypotheses that we tested. For example, in the case of a two species hypothesis (SP14.1), we used species designation based on geographic locations proposed by Spinks et al. (2014) and presence/absence of inguinal scutes. In each case, we were testing to determine if our multivariate data best matched the proposed species/population bins for our different biogeographic hypotheses.

To conduct our ML analyses, we used the R package assignPOP (Chen et al. 2018). The purpose of assignPOP is to use supervised MLMs to assign individuals to populations, both in a training and predictive fashion. Two cross-validation approaches are used within assignPOP, a K-Fold cross-validation (in our case four- or five-fold validations, depending on the number of potential groups included in the training datasets) and a Markov-Chain cross-validation (using 50, 70, and 90% of individuals randomly assigned 30-times each). Each cross-validation technique produces slightly different results. Therefore, for each binning scheme, we ran all five MLMs through both cross-validation schemes. Cross-validation is used to gauge model performance on training datasets as a way of evaluating how the models and/or assignment schemes should work on predictive (non-training data) e.g., if training performance is high, predictive results are expected to be accurate, but if training assignment accuracy is low, predictive results are anticipated to be inaccurate (Chen et al. 2018). Although cross-validation is accepted as a valid approach to evaluate model performance, acceptable output accuracy is subjective and left to the end user to determine. Here, we chose to not evaluate predictive performance of models with assignment accuracy below 95%, based on the generally accepted 95% confidence interval within biostatistics.

## Results

### Morphometrics

**Fig. 3:**
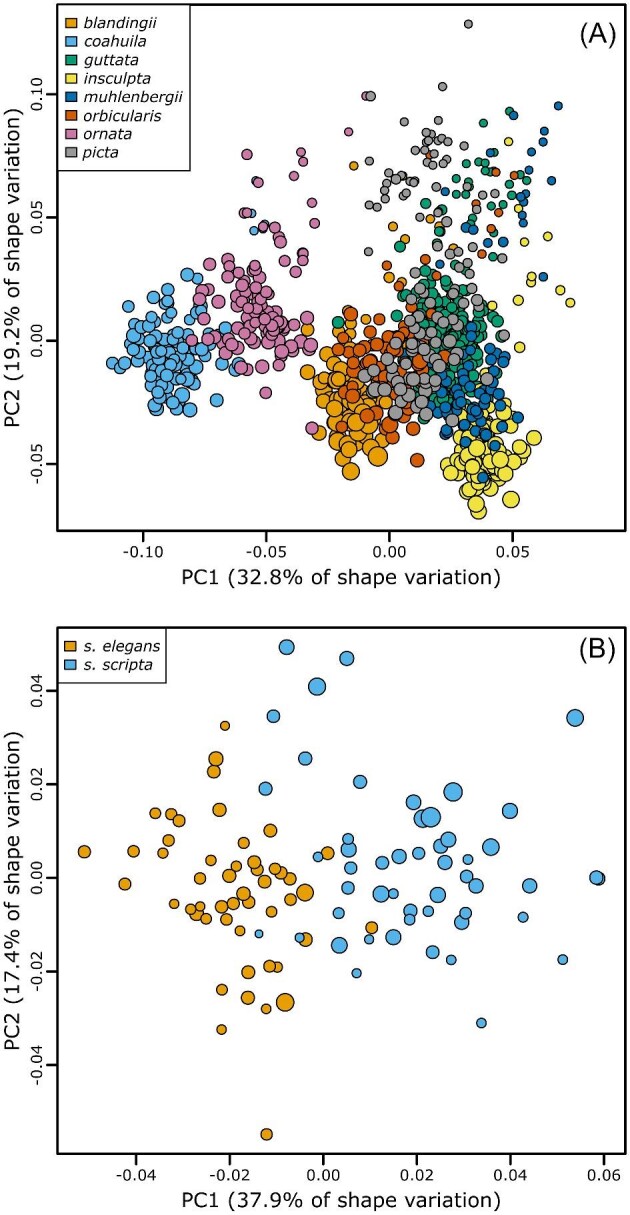
Principal components analysis scatterplots summarizing plastron shape variation in two of the datasets used in this study. (A) Scatterplot of the first two PCA axes from the eight emydid species datatset. (B) Scatterplot of the first two PCA axes from the *Trachemys* subspecies dataset. There are clear distinctions between the different species or subspecies in both datasets. Point colors correspond to the categories within each dataset; point size is proportional to individual centroid size. Parenthetical values in axis labels are the percentage of total variance accounted for by the axis.

**Fig. 4:**
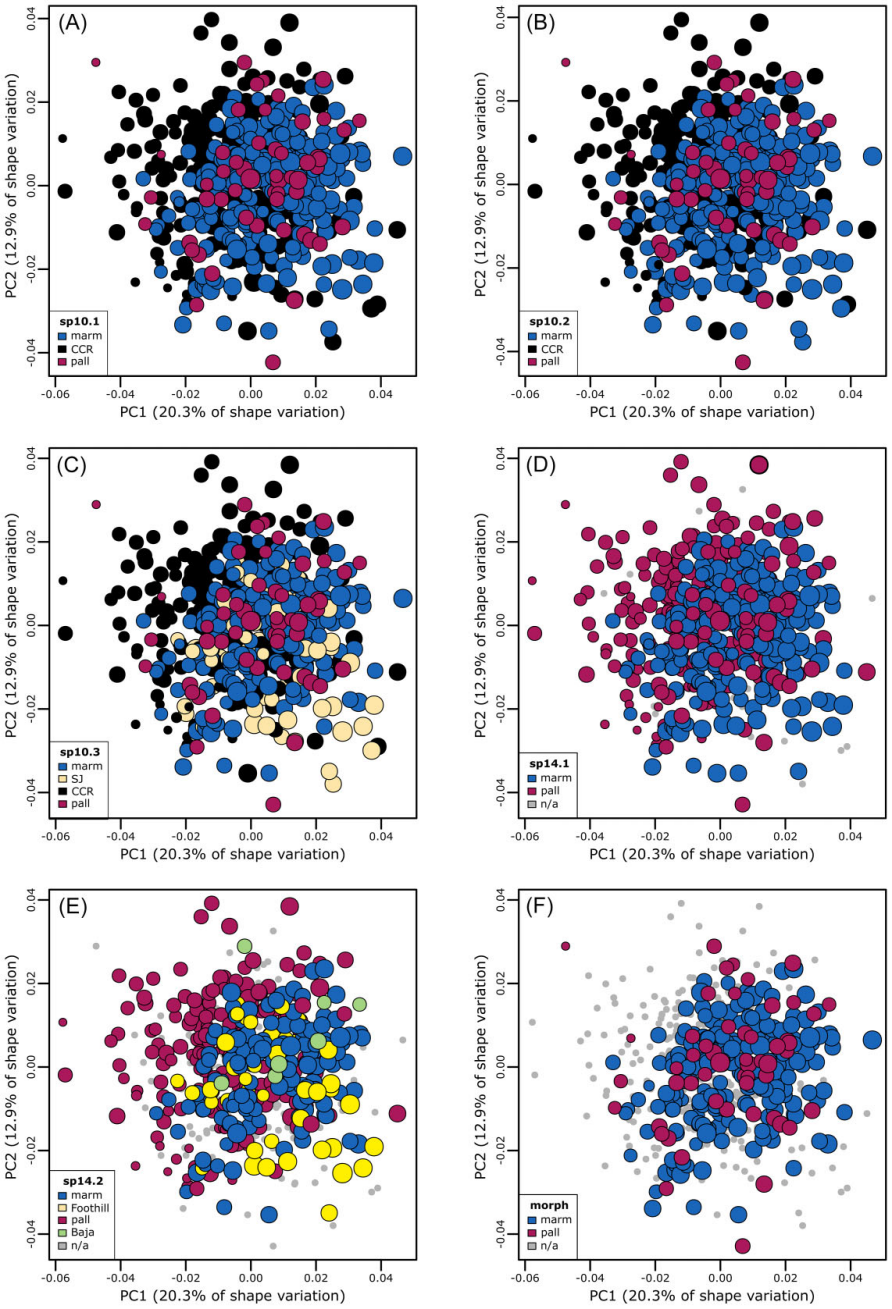
Principal components analysis scatterplots summarizing plastron shape variation in the *A. marmorata* dataset. Each panel corresponds to one of the six different classification schemes analyzed in this study (Table 1). Point color corresponds to categories within each scheme and class names correspond to geographic regions. “N/A” specimens could not be assigned confidently to a class in given scheme. Point size is proportional to individual centroid size, except for N/A specimens. Parenthetical values in axis labels are the percentage of total variance accounted for by the axis.

The results of the PCA of plastron shape in both the eight species (“emydines”) and *Trachemys* datasets demonstrated strong association between shape and the recognized classification schemes (Fig. 3). The results of the PCA of plastron shape in the *A. marmorata* dataset showed no clear connection between plastron shape and any of the proposed classification schemes (Fig. 4). The first PC axis of shape variation appeared to be primarily structured by differences in individual centroid size (Fig. 4). A linear regression of PC 1 versus log centroid size showed that size explained approximately 81% of the variation along this axis. Even though we excluded hatchlings and small juveniles from the dataset, there is a strong pattern of size-related shape variation in the plastron of *A. marmorata* and other emydines (Angielczyk and Sheets 2007; Angielczyk and Feldman 2013) and there was sufficient size variation among the larger individuals for some of this signal to remain in the dataset. This observation was the motivation for including centroid size and its interaction with PC1 as predictors in all of the supervised learning models. Size typically explains <3% of the variance of the remaining PC axes (e.g., 2.5% on PC2).

### Digitization error

Using a Procrustes-ANOVA, we calculated the sum of squares (0.00000606), mean of squares (0.0000000399); and Goodall's F-Statistic (non-rational number). Goodall's *F*-Statistic is the ratio between explained (between-group) and unexplained (within-group) variation in Procrustes distances (Webster and Sheets  2010); a non-rational Goodall's *F*-Statistic indicates within-group variation in Procrustes distances is zero. Taken together, we determined that virtually no variation exists between the different landmark digitization efforts and that error due to digitization is minimal.

### Sexual dimorphism

When all *A. marmorata* specimens of known sex are compared, females have a slightly larger mean centroid size than males, but the means (two-sample *t*-test *P* = 0.4869) and distributions (Kolmogorov–Smirnov *P* = 0.5834) of centroid size do not differ significantly between the sexes. Analysis of the differences between sexes of *A. marmorata* indicated that sex does not appear to strongly structure or bias differences in shape. There is no significant difference in mean shape (Hotelling's *T*-Squared *P* = 0.5806), and no significant difference in distance between group means (Mahalanobis distance *P* = 0.7169). The results from sex comparisons within each classification scheme are similar, with poor general performance of sex as a classifying variable. Taken together, these results indicate that sex is unlikely to be a strong biasing factor in our analyses.

### Inguinal scales

Unlike the case for sex, we observed shape variation associated with the presence or absence of inguinal scales. We recovered significant differences in mean shape between individuals with and without inguinal scales (Hotelling's *T*-squared *P* = 0.001), and a significant difference in distance between those means (Mahalanobis distance *P* = 0.001).

### Habitat variables

**Fig. 5:**
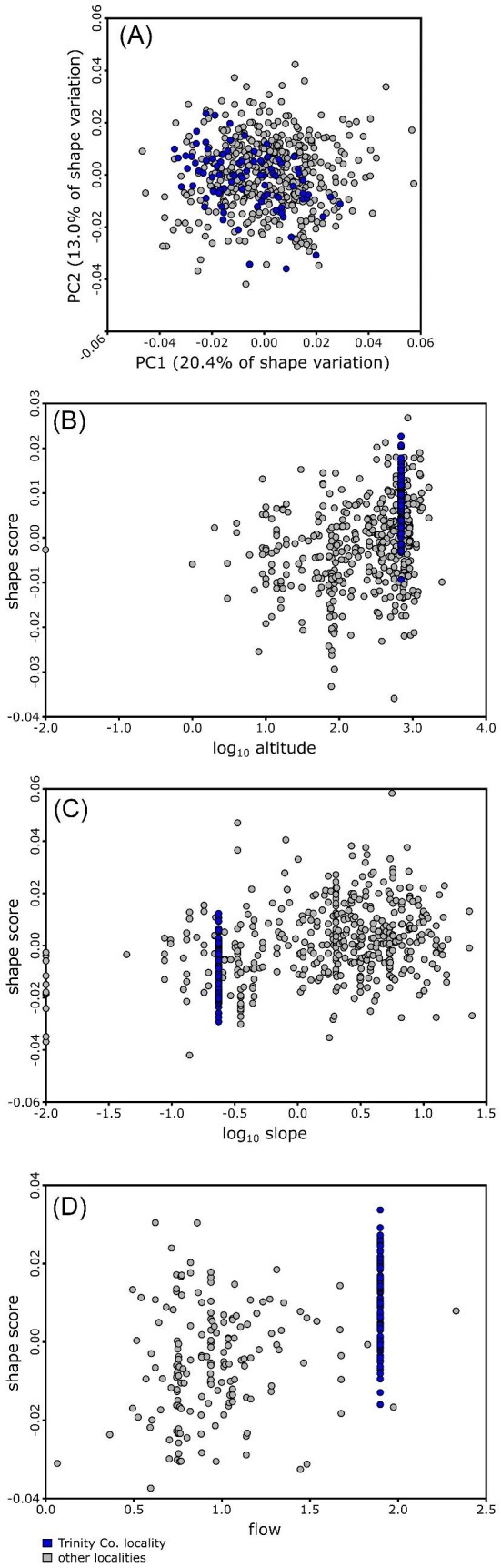
Scatterplots comparing morphological variation present in a single well-sampled locality (89 specimens; Trinity County, CA, USA) to the remainder of the *A. marmorata* dataset. (A) Scatterplot of the first two principal component axes for the full *A. marmorata* dataset. (B) Scatterplot of shape scores (sensu Drake and Klingenberg 2008) from a multivariate regression of shape versus log_10_ altitude. (C) Scatterplot of shape scores from a multivariate regression of shape versus log_10_ slope. (D) Scatterplot of shape scores from a multivariate regression of shape versus flow rate. Specimens from the Trinity County locality are shown in blue; specimens from other localities in gray.

The multivariate regressions showed that slope, altitude, and flow rate were not strong predictors of plastron shape. Of the regressions, only the regression of shape versus log altitude produced a significant result (*P* = 0.0348), and all three models had very low explanatory power (percentage variance explained ranged from 0.21 to 0.72%). Exploration of these results revealed that a subset of 89 specimens from the Museum of Vertebrate Zoology (University of California, Berkeley, CA, USA) collected at a single locality in Trinity County, California, encompassed a similar amount of shape variation as the rest of the dataset (Fig. 5). Because these specimens all have the same associated altitude, slope, and flow rate values, yet display such wide variation in plastron shape, they caused the regressions to have very low explanatory power. At the same time, they underscored the apparently weak impact of the habitat variables on plastron shape.

### Supervised machine learning

Analysis of the eight morphologically and genetically distinct species (“emydines”) and the *T. s. scripta*–*T. s. elegans* (*Trachemys*) datasets indicated that these taxa can be readily differentiated on the basis of plastron shape. Our “emydines” dataset had excellent training performance with cross-validation accuracy above 95% and several models approaching 99% (Table 4, Supplementary Fig. 1). The *Trachemys* dataset performed less well than “emydines,” with cross-validation accuracy above 90% and several models performing above 95% (Table 4, Supplementary Fig. 2). These results demonstrate that when there are distinctions between the states of the classification schemes (i.e., differences in plastron shape that correlate with the different taxonomic groups), the methods used here can recover them.

**Table 4:** Average classification rate of training datasets from machine learning analyses. Best performing models for each cross validation approach are in bold. kf, K-Fold; mc, Markov chain; LDA, linear discriminant analysis; nB, naiveBayes; rf, random forests; SVM, support vector machine; TREE, decision tree classification.

MLA	Emydines	*Trachemys*	SP10.1	SP10.2	SP10.3	SP14.1	SP14.2	Morpho
kf LDA	0.980	0.925	0.586	0.72	0.680	0.830	–	–
mc LDA	0.975	0.905	0.600	0.733	0.677	0.837	–	0.797
kf nB	0.905	0.915	0.558	0.626	0.570	0.758	0.660	–
mc nB	0.910	0.945	0.570	0.628	0.563	0.757	0.670	0.768
kf rf	0.970	0.930	0.583	0.690	0.630	0.828	0.720	–
mc rf	0.965	0.945	0.580	0.683	0.637	0.810	0.717	–
kf SVM	0.970	0.940	0.580	0.718	0.670	0.816	0.734	–
mc SVM	0.975	0.960	0.607	0.733	0.660	0.760	0.730	0.768
kf TREE	0.850	0.808	0.513	0.591	0.516	0.816	0.613	–
mc TREE	0.860	0.810	0.500	0.593	0.493	0.710	0.612	0.767

In contrast, the training performance associated with the *Actinemys* data was generally poor, regardless of classification scheme used. In general, all MLMs performed similar to one another and classification rates were similar between both cross-validation approaches, with the differences being primarily between classification schemes (Table 4; Supplementary Figs. 3–8). Schema SP10.1 had an average classification rate of 58% and a range of 50–64% classification accuracy depending on model and cross-validation approach (Table 4, Supplementary Fig. 3). Schema SP10.2 had an average classification rate of 67.8% and a range of 60–74% classification accuracy (Table 4, Supplementary Fig. 4). Schema SP10.3 had an average classification rate of 61.6% and a range of 50–69% classification accuracy (Table 4, Supplementary Fig. 5). Schema SP14.1 had an average classification rate of 80.5% with a range from 72 to 85% classification accuracy (Table 4, Supplementary Fig. 6). Schema SP14.2 had an average classification rate of 71% and a range of 62–78% classification accuracy (Table 4, Supplementary Fig. 7). Schema Morpho had an average classification rate of 77% with a range of 76–78% classification accuracy (Table 4, Supplementary Fig. 8). We note that for schema SP14.2, only four models with their respective cross-validation schemes (eight analyses in total) could be run compared with the 10 analyses for SP10.1, SP10.2, SP10.3, and SP14.1, and for Morpho only three models (six analyses total) could be run. In both cases, there were errors associated with running cross validations for the LDA model for those classification schema; the additional drop for Morpho came from not being able to run K-Fold cross-validation for the SVM model. Cross-validations for machine learning can fail when sample sizes become small (Varoquaux 2018) or due to overfitting of models to the relative underlying data (Han and Jiang 2014). Because none of our models performed well in training datasets overall, we did not further investigate whether it was sample size, overfitting, or a combination of both which resulted in failures of our cross-validations.

## Discussion

Molecular systematics has repeatedly demonstrated the existence of cryptic species that are more readily diagnosed using genetic data than morphological data (e.g., Stuart et al. 2006; Bickford et al. 2007; Pfenninger and Schwenk 2007; Schlick-Steiner et al. 2007; Clare 2011; Funk et al. 2012; Papenfuss and Parham 2013; Mrinalini et al. 2015; Murphy et al. 2015; Camp and Wooten 2016; Turvey et al. 2019; Chornelia et al. 2022; McGuire et al. 2023). Because of this ability to readily diagnose species, and attempts to streamline the documentation of biodiversity, several methods of species delimitation that rely almost entirely on genetic data have been proposed (e.g., Pons et al. 2006; Carstens and Dewey 2010; Hasdorf and Hennig 2010; O'Meara 2010; Yang and Rannala 2010; Huelsenbeck et al. 2011; Dufresnes et al. 2023). Although strong caveats on the utility of these methods have been raised (e.g., Bauer et al. 2010; Carstens et al. 2013; Zachos et al. 2013; Sukumaran and Knowles 2017; Stuck et al. 2017), they are nevertheless being used to name species (e.g., Leaché and Fujita 2010; Spinks et al. 2014). In contrast to those genetically diagnosed species, the majority of extant taxa and almost all extinct taxa are delimited by morphology alone. This disjunction complicates interpretations of variation and taxonomic diversity in deep time (e.g., Gould and Eldredge 1977). Because morphological stasis may mask true underlying diversity (Eldredge and Gould 1972; Gould and Eldredge 1977; Van Boxclaer and Hunt 2013), the existence of and difficulty in diagnosing cryptic species using morphology also has serious implications for our records of modern biodiversity preserved in natural history collections. Despite recent methodological advances for obtaining molecular data from historical specimens, including formalin-fixed museum specimens (e.g., Ruane and Austin 2017), challenges remain in obtaining the genetic data needed for non-morphological species delimitation methods. These considerations emphasize the need to investigate whether geometric morphometric analyses can capture fine-scale variation that can be used for morphologically delimiting cryptic species.

In this study, we tested whether geometric morphometric plastron shape data analyzed with an ensemble of supervised machine learning techniques could differentiate species and sub-species of emydid turtles. We first validated the methods with two datasets involving groups whose taxonomy is uncontroversial: a series of eight morphologically and genetically distinct emydid species with disparate plastron shapes, and two subspecies of *T. scripta* that are primarily differentiated on the basis of geographic range and color variation (Bonin et al. 2006). Our results yielded high out-of-sample classification performance for these datasets. The results indicated that when even subtle class separation in plastron shape exists, our approach was able to detect it and make good out-of-sample predictions.

Following validation of our approach, we applied it to a more controversial example: morphologically diagnosing the putative cryptic species *A. marmorata* and *A. pallida* (sensu Spinks et al. 2014). Our results for this system show that the two best-performing hypotheses for the *A. marmorata* complex are Morpho (best performing model 79.7% classification rate) and SP14.1 (best model 83.7% classification rate). Both differentiated *A. marmorata* and *A. pallida* from one another. However, it should be noted that although these models performed better than all other classification schemes, their performance was still well below those recovered for our *Trachemys* and “emydine” validation datasets. Overall, poor performance was a hallmark of the *Actinemys* dataset as opposed to an aberration. Ideal performance of MLMs would show a low spread of variation in cross-validation rates and have classification rates in training datasets above 95%. In all cases for the *Actinemys* data, no single MLM had a classification rate for training data over 84% and most performed considerably lower than this highmark.

A potential analytical explanation for the poor performance is that the level of digitization error in the *A. marmorata* dataset was so great as to swamp out any biological signal. However, we recovered no evidence of digitization error (see Methods Digitization Error). Likewise, PCA ordinates individuals along axes of maximum variance that need not align exactly with biologically meaningful differences (Polly and Motz 2016), so it is possible that between-group variation may be reflected by differences not recovered in the PC used as predictor variables in our analyses. To interrogate this issue, we treated our aligned Procrustes coordinates as predictor variables within assignPOP analyses. Doing this resulted in consistently poorer performance for the *Trachemys* and *Actinemys* datasets, with a reduction in accurate assignment of nearly 10% for *Trachemys* and a reduction of more than half for *Actinemys*, dropping below random (50%) to 25% accuracy. These results indicate that dimensional reduction from PCA served to ordinate our data in a way that makes it easier to recover whatever signal is present instead of obscuring it.

Perhaps more problematic are the low sample sizes available for some of our subgroups, especially the “Baja” subgroup in SP14.2. Low sample sizes reduced the power of the MLMs we employed, but we do not think this problem is fatal because other *A. marmorata* grouping schemes with subgroups characterized by larger sample sizes (e.g., SP10.1) did not perform dramatically better. Although we would like to have additional samples for our analyses, given our thorough sampling of available museum collections of *A. marmorata*, increasing available sample sizes likely would require additional fieldwork to photograph and/or collect new specimens.

Alternatively, there may be a real biological shape variation that is overwhelming differentiation associated with genetic divergence. For example, it has been suggested that shell shape in *A. marmorata* differs among populations inhabiting bodies of water with different flow regimes (Holland 1992; Lubcke and Wilson 2007; Germano and Bury 2009). We attempted to account for this in our analyses by examining the relationship between plastron shape and two proxies of flow regime (elevation and regional slope) for our complete dataset, as well as the relationship between shape and direct measurements of flow rates for a subset of specimens. In all cases, the resulting regressions had very low explanatory power, and the large sample of specimens from a single Trinity County locality (with identical elevation, slope, and flow values) displayed nearly as much shape variance as the rest of the dataset. Therefore, it seems unlikely that the flow regime significantly biased our results. Sexual dimorphism also does not appear to introduce bias to our dataset, as there is no significant difference in mean shape between males and females. *Actinemys marmorata* simply appears to have a highly conservative plastron shape, as evidenced by the fact that the species as a whole shows about as much variance as our large single locality population.

It is important to underscore that plastron shape is an effective method for differentiating classes in the other datasets we investigated (*Trachemys* and the “emydines”), which represent both more ancient and similarly recent and likely incomplete divergences as the *Actinemys* case (Spinks et al. 2016; Parham et al. 2020; Vamberger et al. 2020). The magnitude of shape differences between the eight species (measured as Procrustes distance between the eight species’ mean shapes) is approximately an order of magnitude greater than the differences between the *A. marmorata* subgroups, and not surprisingly the MLMs had no trouble classifying those specimens correctly. However, the magnitude of the shape differences between the *T. scripta* subspecies is comparable to those separating the different *A. marmorata* subgroups, yet even in that case, the MLMs returned an almost perfect classification. These results demonstrate that plastron shape can potentially be a good marker for differentiating real subgroups, thus supporting our contention that the negative results for *A. marmorata* are not simply a shortcoming of the methods we applied. Indeed, it begs the question of what factors have suppressed morphological differentiation of plastron shape in *A. marmorata* and *A. pallida* if they are distinct species. Invoking issues, such as the role of the plastron in protection or the need for streamlining are insufficient because the other species are expected to be subject to similar constraints (Stayton 2011; 2019; Polly et al. 2016; Stayton et al. 2018).

It is possible that our morphometric landmarks simply were inadequate to capture the necessary signal to distinguish between *A. marmorata* and *A. pallida*. The addition of landmark data circumscribing the general outline of the plastron and scale characters may reveal additional between-group distinctions. We are skeptical that such an approach will ultimately prove fruitful for multiple reasons. First, our landmark data consistently recover signal in both distantly related taxa (e.g., “emydines”) and in closely related sub-species (e.g., our *Trachemys* data) facilitating their differentiation using a variety of MLMs. This is in addition to these landmark data having shown to be adequate for revealing fine-scale ontogenetic variation in closely related taxa (e.g., Angielczyk and Feldman 2013). Second, the addition of scute characters is problematic due to the fact that many characters commonly used for phylogenetic distinction, such as the length of scute sutures or the contacts between scutes that form sutures are known to be variable within single, genetically stable, species, such as *Terrapene coahuila* (Burroughs et al. 2013). Indeed, the need to adequately describe variation for potential phylogenetically informative characters within testudinoid turtles is a well-known issue that has plagued turtle systematists for decades (e.g., Hirayama 1984; Gaffney and Meylan 1988; Joyce and Bell 2004). Finally, recent work evaluating the efficacy of general plastral landmarks and shell landmarks for reconstructing phylogeny indicates that such morphometric data generally do not reconstruct better-supported and accepted phylogenetic relationships (Ascurrunz et al. 2019).

Moving beyond the plastron, an interesting direction for future work would be to test whether 3D landmark data collected on the carapace of *Actinemys*, or the whole shell (i.e., the carpace and plastron together), can better differentiate subgroups recognized on the basis of geographic occurrence or patterns of genetic divergence. This approach would be particularly interesting because in some ways it is a more direct parallel to Holland's (1992) work using linear measurement data, and it almost certainly would capture a greater amount of shape variation. However, it would also be more challenging logistically to digitize a comparably large number of specimens in 3D.

### Taxonomic implications

In the time since Spinks et al.’s (2014) publication elevating *A. pallida* to the rank of species, the “two-species” hypothesis has become the standard taxonomic framework for the *Actinemys* complex (e.g., Rhodin et al. 2021). Our results provide some equivocal support for this hypothesis, but plastron shape alone seems to have limited utility at best for diagnosing *A. marmorata* and *A. pallida*. Furthermore, as we discuss below, much of the other data that have been used to justify the separation of the species is similarly inconclusive.

Spinks et al. (2014) elevated *A. pallida* based on a species delimitation analysis of SNP data using BPP (Yang and Rannala 2010). However, Carstens et al. (2013) reviewed the shortcomings of validation methods, such as BPP that rely on guide trees and concluded that they should be interpreted with caution. The mitochondrial data from Spinks et al. (2014) show extensive introgression and admixture in Central California. This pattern is likely not just a mitochondrial sweep because there are no significant physical barriers to nuclear gene flow in this region. They also lack samples from populations in the San Francisco Bay Area, which we predict would likely show even more genetic mixing. Carstens et al. (2013) strongly emphasized that inferred species boundaries based only on genetic data will likely be inadequate, and that morphological, behavioral, geographic, and life history data also should be incorporated into the process of diagnosing species as much as possible. These caveats evoke the development of the Unified Species Concept (Dayrat 2005; De Queiroz 2007), Integrative Taxonomy (Padial et al. 2010), and other pluralist approaches to species delimitation. The apparent complexity of the *Actinemys* example, combined with the well-demonstrated ability for testudinoid turtles to hybridize (e.g., Buskirk et al. 2005; Spinks and Shaffer 2009; Parham et al. 2013) and the fairly high levels of geographically structured genetic variation in its closer relatives (Amato et al. 2008; Davy and Murphy 2014; Sethuraman et al. 2014), underscore the importance of such approaches.

From a morphological perspective, Spinks et al. (2014) cited Seeliger's (1945) range-wide examination of morphological variation in *A. marmorata* as being consistent with their results, but there are problems with that claim. For example, Spinks et al. (2014) stated that Seeliger indicated that extensive intergradation was limited to the San Joaquin Valley, but Seeliger also hypothesized that her concept of *C. m. pallida* and *C. m. marmorata* intergrade in the vicinity of the San Francisco Bay (Coast Ranges) and listed localities in Alameda, Contra Costa, and Santa Clara counties where this occurs. Spinks et al. (2014) lacked samples from the Bay Area, but their closest specimens to the east showed signs of intergradation. Our large sample size may have recovered a greater signal of variation representative of hybridization than recovered in the SNP analyses of Spinks et al. (2014), leading to less clear-cut boundaries between populations in our results. Furthermore, Seeliger's (1945) morphological characters themselves are problematic. Issues with the presence/absence of inguinal scales were discussed above, and coloration of the neck (the other main diagnostic character) is also quite variable, especially in areas of proposed intergradation (Seeliger 1945; Buskirk 1990).

In this work, we have largely followed the “two-species” framework for *Actinemys* taxonomy, but in light of the various issues noted above, we consider this more of a working hypothesis than a robust, settled taxonomic conclusion. Given the complexities of this system, the fact that our morphometric data and machine learning analyses were able to recover any signal consistent with the existence of two species is impressive, and speaks to the effectiveness of the methods that was evidenced in the results from the emydine and *Trachemys* validation analyses. We fully agree with Spinks et al. (2014) that *A. marmorata* sensu lato is a species deserving of strong conservation efforts, and the genetic diversity uncovered by the analysis of Spinks et al. (2014) should be accounted for explicitly in any conservation plans (e.g., U.S. Fish and Wildlife Service 2023). Yet, we also underscore the need for additional morphological and molecular work on *Actinemys* to further refine our knowledge of its evolutionary history and phylogeographic structure, which in turn can form the basis for informed taxonomic and conservation decisions.

## Conclusions

We used a combination of geometric morphometrics and supervised MLMs to test whether plastron shape can differentiate the *A. marmorata* and *A. pallida*. Validation of the method using data for eight well-established emydid species and two sub-species of *Trachemys scripta* showed that plastron shape is typically highly effective at differentiating taxonomic groups of interest. By contrast, this approach was less successful at differentiating between various taxonomic hypotheses for the *A. marmorata* complex: most classification models performed poorly, although the two best-performing models provided weak support for a distinction between *A. pallida* and *A. marmorata*. Plastron shape in the *A. marmorata* complex does not appear to be strongly correlated with flow regime or its proxies (elevation, regional slope). Despite these results and problems with the genetic data used to justify the separation of *A. marmorata* and *A. pallida*, we suggest that the elevation of *A. pallida* to species status is a suitable working hypothesis for future research. However, the diagnoses for the two species will likely continue to be complicated by a significant range of intergradation.

Although it is tempting to conclude that the use of MLMs for hypothesis-driven biogeographical studies and taxonomic questions is inappropriate because our MLMs performed more poorly in *Actinemys* than they did our in emydine and *Trachemys* datasets, we do not agree with such a view. *Actinemys marmorata* represents a very complex biogeographical history and, despite low amounts of variation in our morphological traits, we still detected some (albeit low) signal. That the same MLMs work exceedingly well on other taxa indicates their potential utility and we encourage other researchers to use them to explore their datasets with some caveats. First, MLMs appear to work best in traits with higher amounts of variation, and geometric morphometric data suits this criterion well. Second, although it is clear that MLMs can be used to evaluate different biogeographic and taxonomic hypotheses, these hypotheses must still be formed *a priori* based on other observations. Because supervised MLMs do not require a specific underlying format to the input data, almost anything can be used as an input. Thus, it is imperative that the end user make sure that the input data is being used to investigate explicit hypotheses.

With this in mind, we can answer our title question, “Can machine learning aid in cryptic species identification?” Yes, supervised MLMs can aid in cryptic species identification, but they have less utility for data exploration or as confirmatory tools. A wide range of potential model performances and relatively subjective assessments of model performance means that it is important to view MLMs as tools for hypothesis testing, and our conclusions must be based not only on MLM support, but on additional evidence as exemplified within this study.

## Supplementary Material

obae010_Supplemental_Files

## Data Availability

The data underlying the article are available in the Dryad Digital Data Repository, at: https://doi.org/10.5061/dryad.wm37pvmv1.
