# Supplementary figures and images for: Morphological Species Delimitation in The Western Pond Turtle (Actinemys): Can Machine Learning Methods Aid in Cryptic Species Identification?
Source: Integr Org Biol. 2024 Apr 2;6(1):obae010. doi: 10.1093/iob/obae010 (PMC11058871; doi:10.1093/iob/obae010)

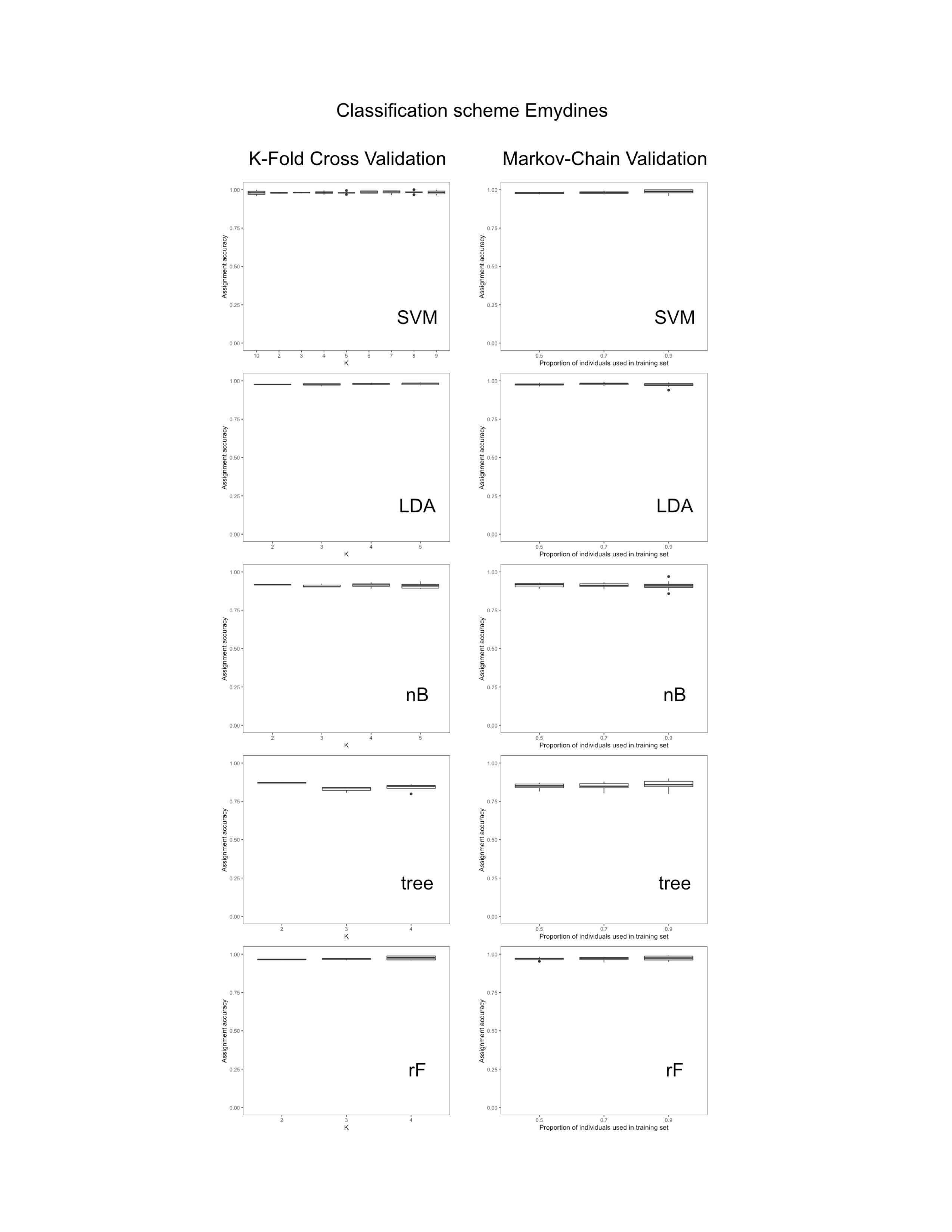

Supplement: obae010_Supplemental_Files [file obae010_supplemental_files.zip › Supp_Fig1_Emydines.tiff]

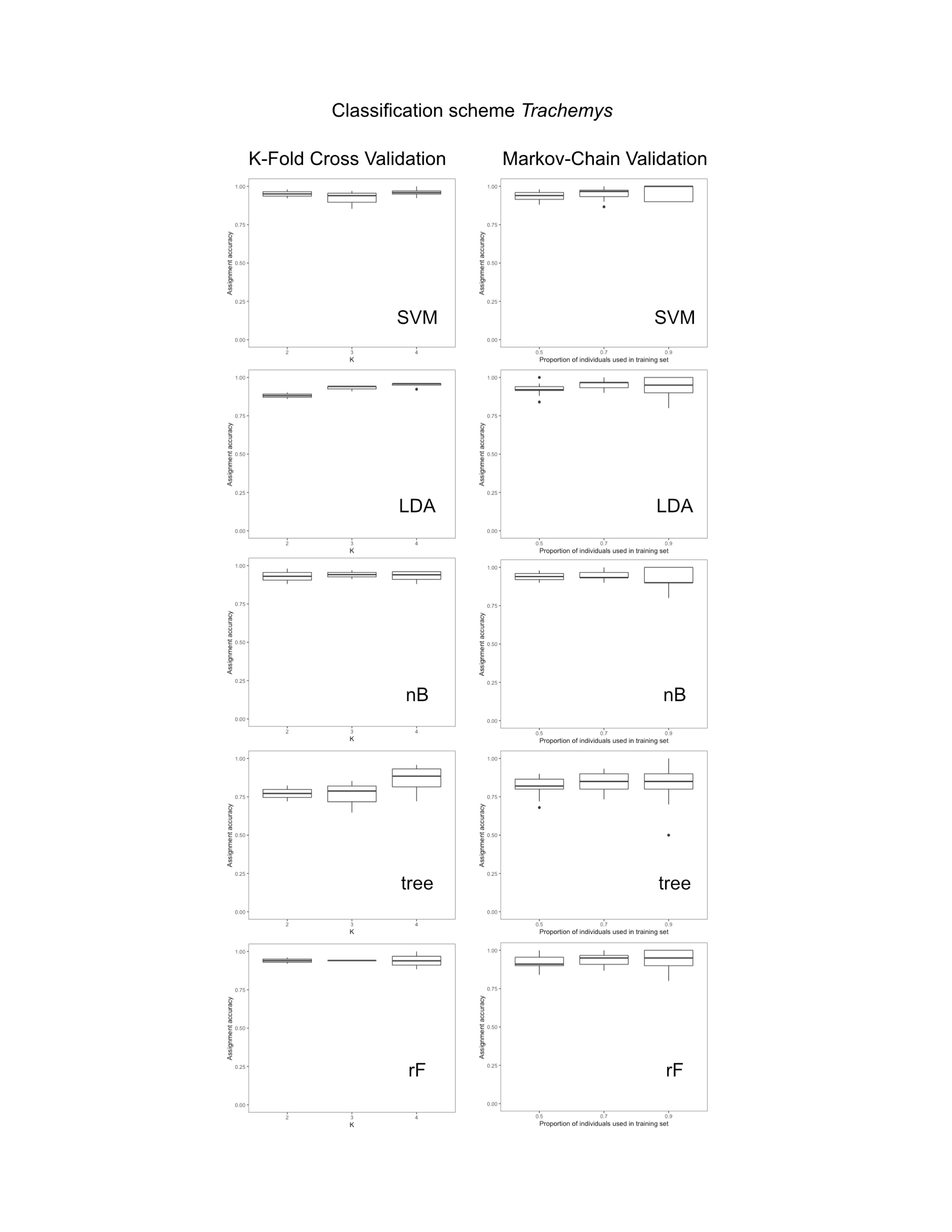

Supplement: obae010_Supplemental_Files [file obae010_supplemental_files.zip › Supp_Fig2_Trachemys.tiff]

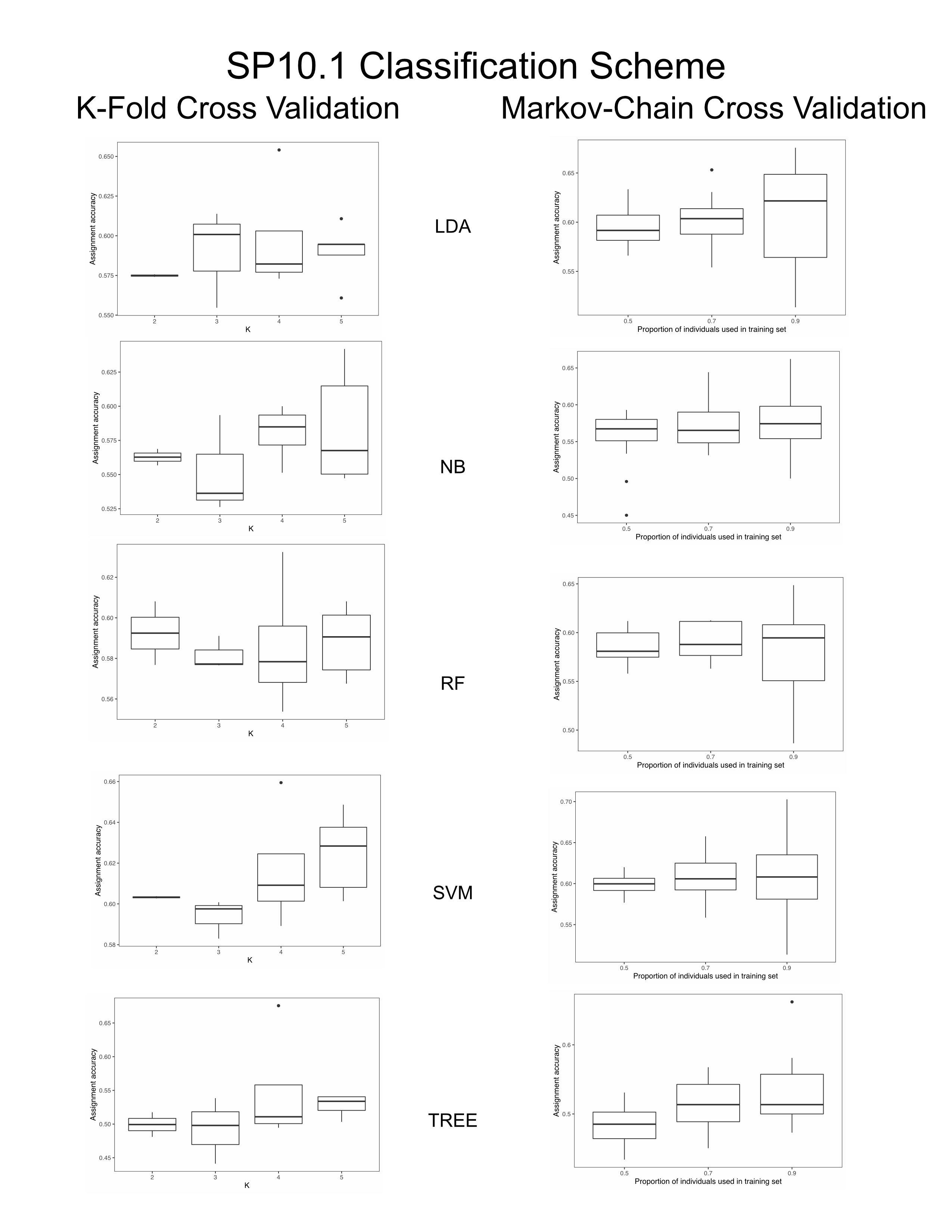

Supplement: obae010_Supplemental_Files [file obae010_supplemental_files.zip › Supp_Fig3_SP10.1.tiff]

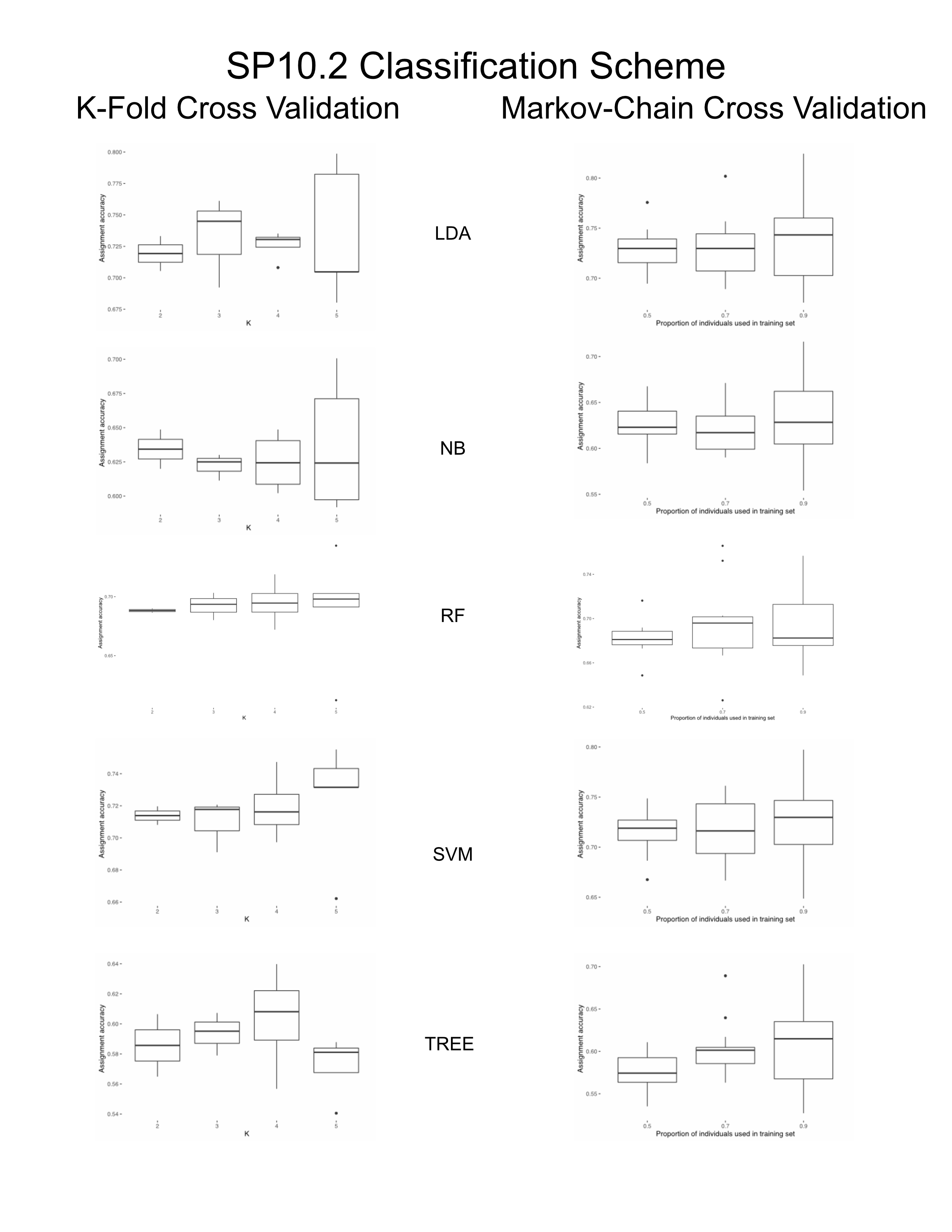

Supplement: obae010_Supplemental_Files [file obae010_supplemental_files.zip › Supp_Fig4_SP10.2.tiff]

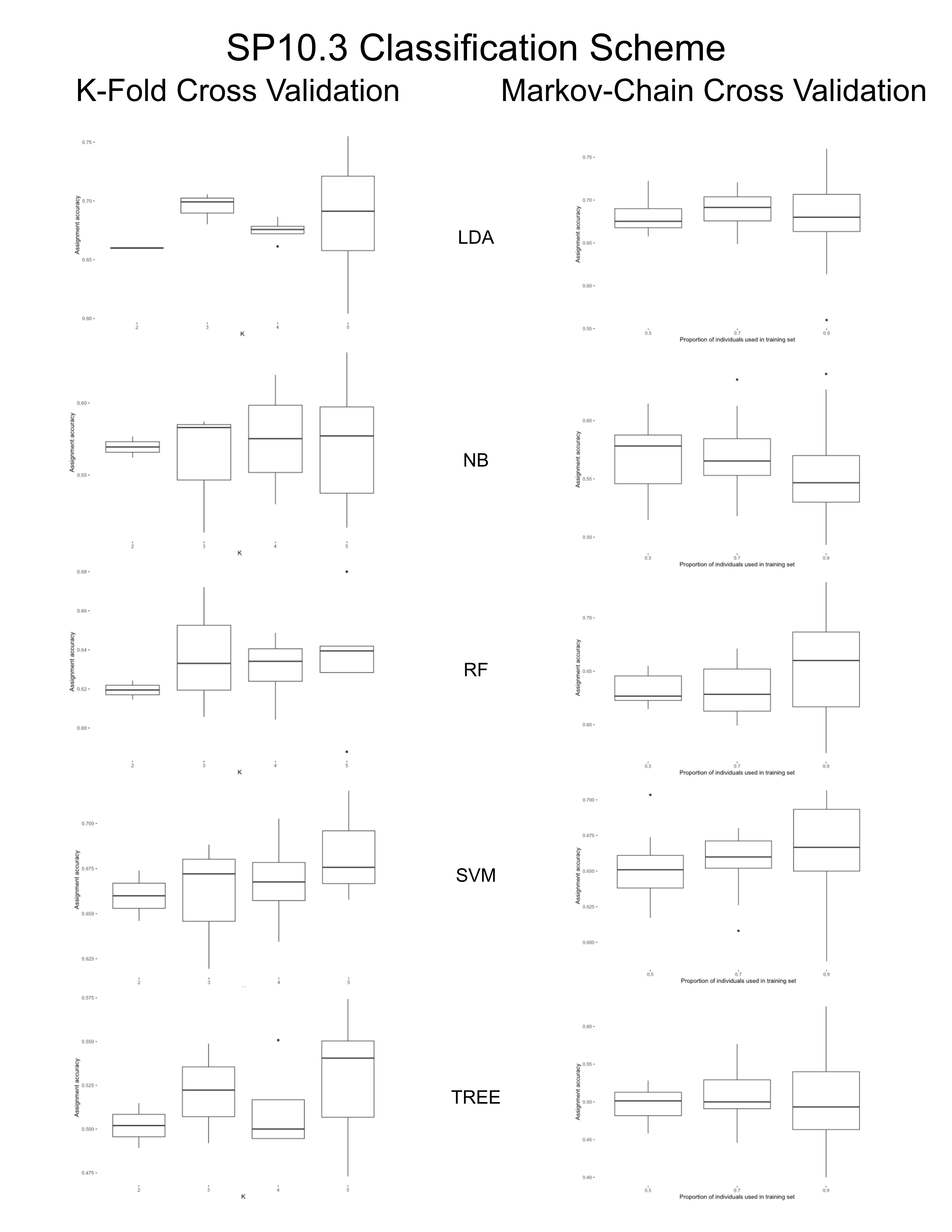

Supplement: obae010_Supplemental_Files [file obae010_supplemental_files.zip › Supp_Fig5_SP10.3.tiff]

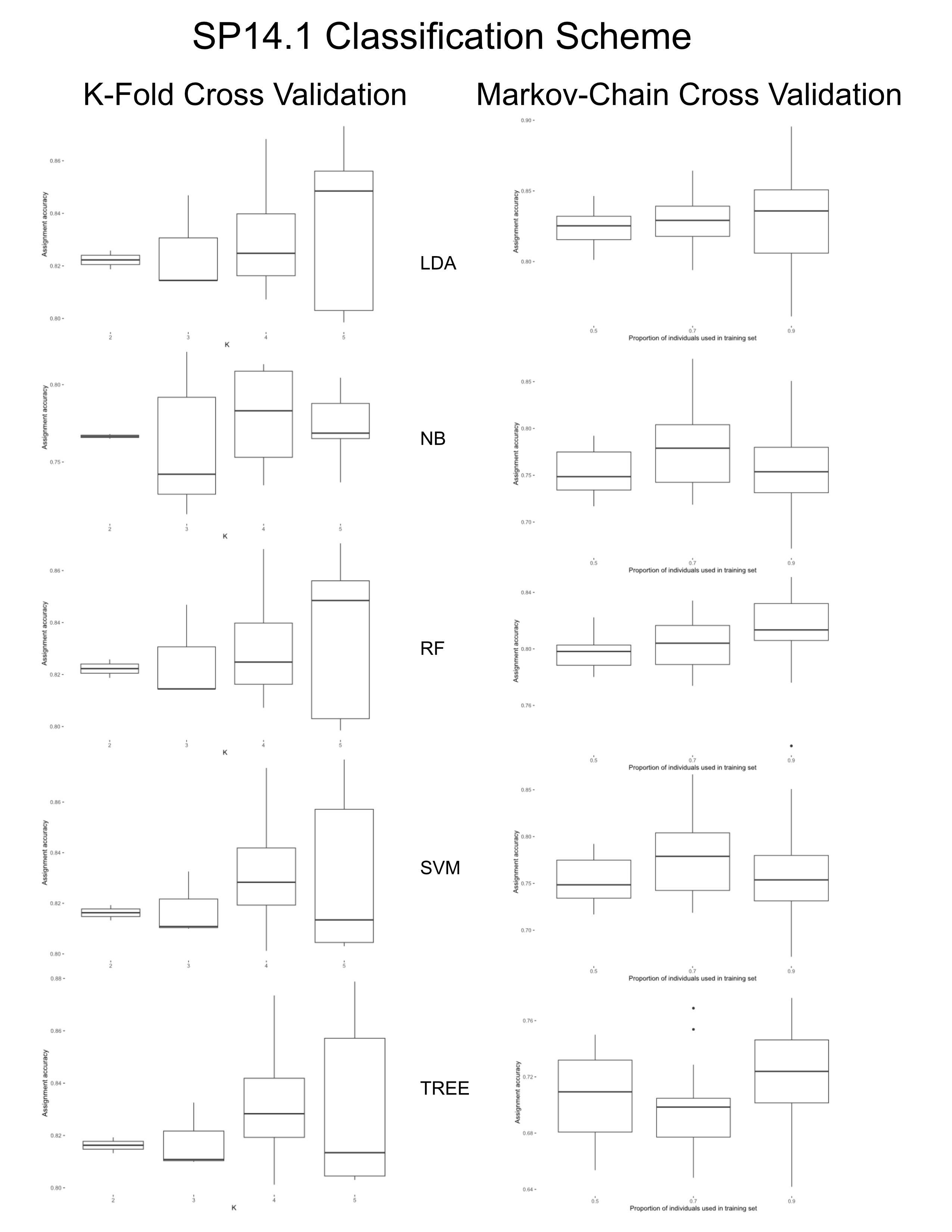

Supplement: obae010_Supplemental_Files [file obae010_supplemental_files.zip › Supp_Fig6_SP14.1.tiff]

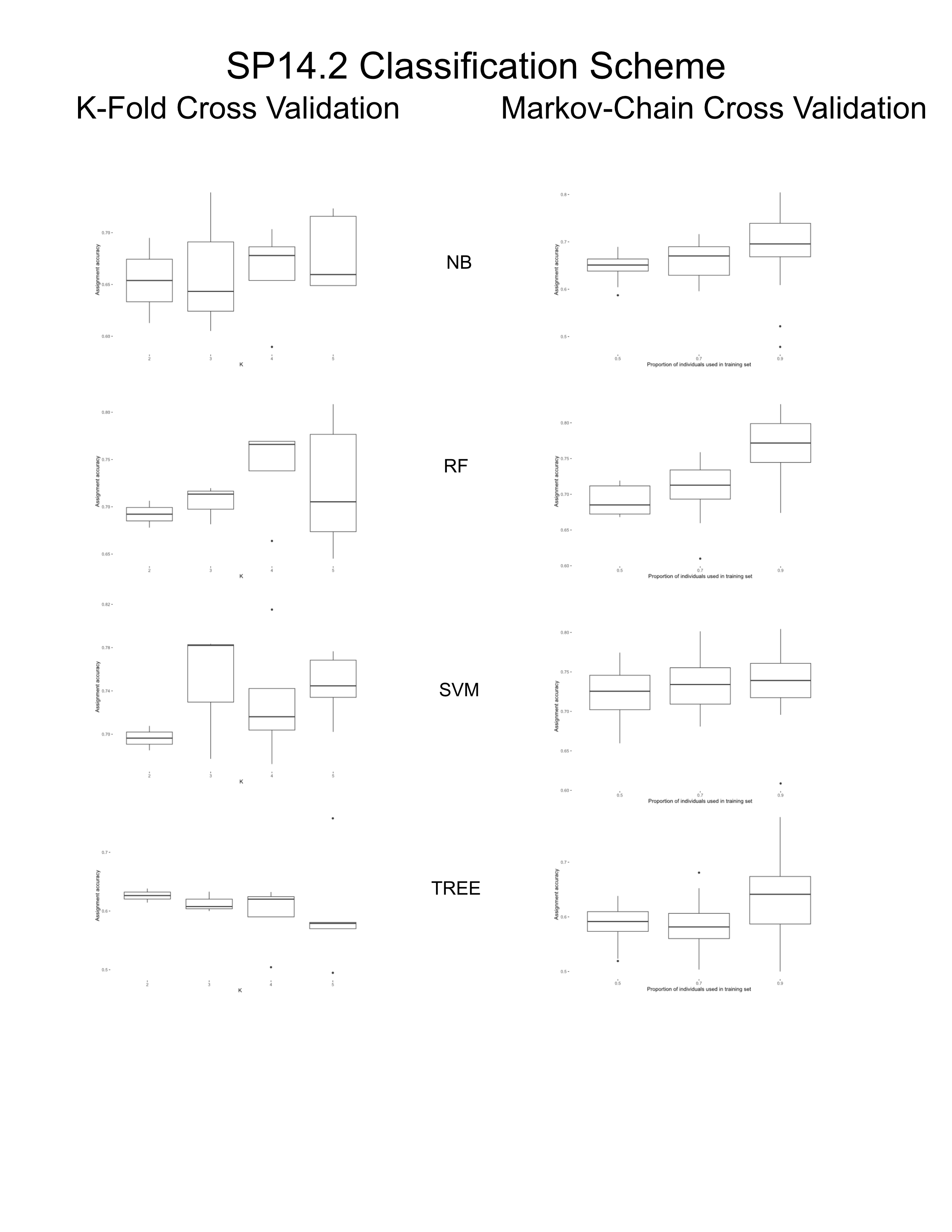

Supplement: obae010_Supplemental_Files [file obae010_supplemental_files.zip › Supp_Fig7_SP14.2.tiff]

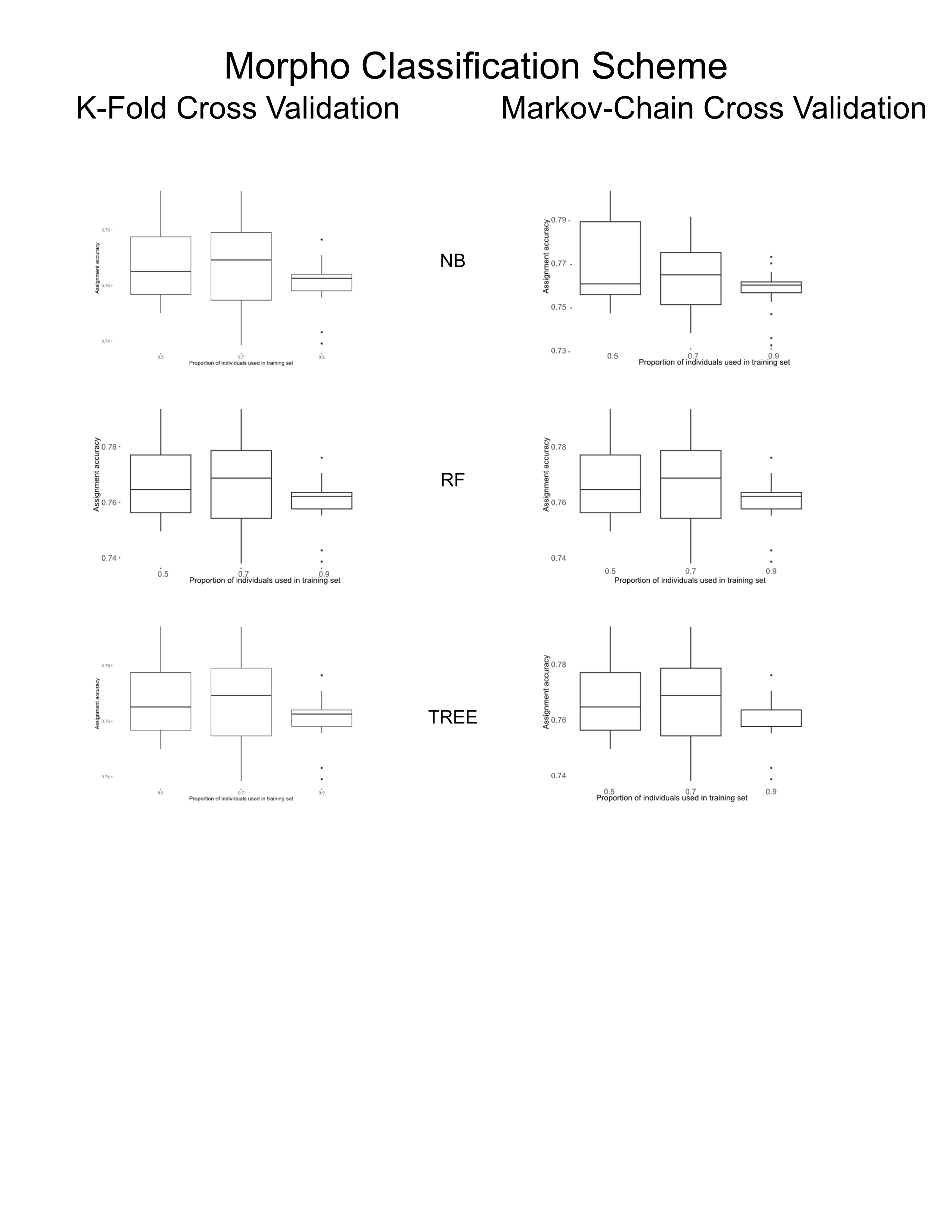

Supplement: obae010_Supplemental_Files [file obae010_supplemental_files.zip › Supp_Fig8_Morpho.tiff]
